# Blood Flow Restriction During Futsal Training Increases Muscle Activation and Strength

**DOI:** 10.3389/fphys.2019.00614

**Published:** 2019-05-22

**Authors:** Sadegh Amani-Shalamzari, Farid Farhani, Hamid Rajabi, Ali Abbasi, Ali Sarikhani, Carl Paton, Mahdi Bayati, Daniel Berdejo-del-Fresno, Thomas Rosemann, Pantelis Theodoros Nikolaidis, Beat Knechtle

**Affiliations:** ^1^Department of Exercise Physiology, Faculty of Physical Education and Sports Sciences, Kharazmi University, Tehran, Iran; ^2^Department of Biomechanics and Sports Injuries, Faculty of Physical Education and Sports Science, Kharazmi University, Tehran, Iran; ^3^Faculty of Health and Sport Science, Eastern Institute of Technology, Napier, New Zealand; ^4^Department of Exercise Physiology, Sports Medicine Research Center, Sport Sciences Research Institute, Tehran, Iran; ^5^Head of Performance at Bay Area Futsal Club, San Francisco, CA, United States; ^6^Institute of Primary Care, University of Zurich, Zurich, Switzerland; ^7^Exercise Physiology Laboratory, Nikaia, Greece; ^8^Medbase St. Gallen Am Vadianplatz, St. Gallen, Switzerland

**Keywords:** small sided game, peak torque, insulin growth factor-1, myostatin, electromyography

## Abstract

The aim of this study was to investigate the effect of leg blood flow restriction (BFR) applied during a 3-a-side futsal game on strength-related parameters. Twelve male futsal players were randomly assigned into two groups (*n* = 6 for each group) during 10 training sessions either with or without leg BFR. Prior to and post-training sessions, participants completed a series of tests to assess anabolic hormones and leg strength. Pneumatic cuffs were initially inflated to 110% of leg systolic blood pressure and further increased by 10% after every two completed sessions. In comparison with baseline, the resting post-training levels of myostatin (*p* = 0.002) and IGF-1/MSTN ratio (*p* = 0.006) in the BFR group changed, whereas no change in the acute level of IGF-1 and myostatin after exercise was observed. Peak torque of knee extension and flexion increased in both groups (*p* < 0.05). A trend of increased neural activation of all heads of the quadriceps was observed in both groups, however, it was statistically significant only for rectus femoris in BFR (*p* = 0.02). These findings indicated that the addition of BFR to normal futsal training might induce greater neuromuscular benefits by increasing muscle activation and augmenting the hormonal response.

## Introduction

Futsal is an intermittent high-intensity game with many resting intervals, e.g., 75% of the playing actions last 1–18 s, whereas more than 83% the resting intervals are 1–15 s (Barbero-Álvarez et al., 2004). Although the scheduled time of the game is 40 min, the duration may last more than 75 min (Naser et al., 2017). Players may cover 3–5 km during a game with more than 50% of the distance covered at a high intensity (>90% of maximal heart rate, HRmax). Previous studies have reported that players achieve blood lactate concentration 5.3 mmol L^−1^ and spend 46% of playing time at exercise intensities higher than 80% of maximal oxygen uptake (VO_2_max; Castagna et al., 2009; Naser et al., 2017). Therefore, futsal played at a professional level is a highly stressful exercise which taxes the zones of explosive (jumping, shooting), aerobic and anaerobic capabilities of players. Thus, futsal players frequently demonstrate VO_2_max values of >60 mL⋅kg^−1^⋅min^−1^ to enable them to cope with physiological requirements of the game (Alvarez et al., 2009; Castagna et al., 2009).

Based on the principle of training specificity, futsal coaches are always looking for more effective training drills. To this end, futsal coaches prefer to use SSG to increase the aerobic and anaerobic capacity of players (Halouani et al., 2014) while simultaneously improving skill. Previous studies supported SSG as an effective training regime compared to generic training (Dellal et al., 2008; Amani-Shalamzari et al., 2019) and reported that SSG was an effective way of improving both aerobic and anaerobic fitness (Impellizzeri et al., 2006). Previous research has reported that 12 weeks of SSG was equally effective at improving aerobic fitness as generic training such as interval running with an intensity of 90–95% of HRmax (Impellizzeri et al., 2006).

Recently several studies have proposed BFR training may improve muscle size and strength, and aerobic capacity even when using low-intensity aerobic training (Abe et al., 2006; de Oliveira et al., 2016). In BFR training, a restrictive device is placed on the proximal end of a limb, reducing the amount of arterial blood flow, and occluding venous return (Pope et al., 2013), resulting in a reduced amount of oxygen supplied to the active muscle. These hypoxic conditions place the muscle under greater metabolite stress by increased lactate and catecholamine concentrations (Tanimoto et al., 2005) which may cause increases in anabolic hormones like GH (Takano et al., 2005), and IGF-1 (Abe et al., 2005), although most studies have demonstrated non-significant findings (Reeves et al., 2006; Loenneke et al., 2011), but Madarame et al. (2010) reported lower body exercise with BFR can acutely increase total testosterone (Madarame et al., 2010). However, research has shown that there is no causal relationship between hormone production and training adaptation, but there is an association between them (Morton et al., 2018).

Development of muscle mass and strength is regulated by both neural and hormonal factors (Marcotte et al., 2015). An increase in muscle strength without hypertrophy indicates greater neural involvement in strength development and maybe inferred with an increase in the amplitude of EMG activity of the muscles. Increased iEMG following low intensities training with BFR has previously been reported (Yasuda et al., 2014; Loenneke et al., 2015), and this increase in iEMG might be associated with increased muscular strength and activation of fast twitch fibers (Yasuda et al., 2014). Endocrine factors also play a positive role in strength acquisition. Growth hormone and IGF-1 may be positive contributors to muscle mass and strength gains by activating the growth signaling pathway of mTOR within muscle fibers (Rommel et al., 2001). Researchers have shown that BFR training induces rapid increase in plasma GH, because of the proposed association between lactate and GH release (Takarada et al., 2000a; Reeves et al., 2006; Patterson et al., 2013). Although GH may not be anabolic *per se*, it stimulates the release of IGF-1 (Kraemer and Ratamess, 2005) and IGF-1 stimulates hypertrophic pathways (Velloso, 2008). A study examining the response of IGF-1 to BFR resistance training on old men reported no change in plasma IGF-1 levels (Patterson et al., 2013), but a study examining the chronic response (two weeks of twice-daily BFR training) on young men showed an increase in basal levels of circulating IGF-1 when compared to baseline (Abe et al., 2005). In contrast, 6 weeks of BFR training reported no changes in baseline levels of circulating IGF-1 (Karabulut et al., 2013). MSTN acts as a negative regulator of muscle mass through suppression of the IGF-mTOR signaling pathway in skeletal muscle (Lee, 2004). In fact, the IGF-1/MSTN ratio may characterize the developmental status of muscle mass (Hu et al., 2015). It has been shown that BFR resistance training can decrease myostatin mRNA when compared to the non-BFR group (Laurentino et al., 2012). Since low-intensity aerobic BFR training may produce small gain in strength and size of muscles (Abe et al., 2006, 2010), it would be interesting to examine acute and chronic response IGF-1 and MSTN to aerobic training with BFR.

Generally, previous research has examined BFR in conjunction with low intensity aerobic and resistance training, whereas few studies have used BFR with higher-load training (Laurentino et al., 2008; Keramidas et al., 2012; Cook et al., 2014; Paton et al., 2017), and the results were somewhat contradictory. Studies by Keramidas et al. (2012) and Laurentino et al. (2008) reported no added benefit on VO_2_max (Keramidas et al., 2012) and muscle strength (Laurentino et al., 2008), respectively following 6–8 weeks of intense training. In contrast, Cook et al. (2014) reported greater improvements in muscle strength compared with non-occluded controls following 3 weeks of strength training (Cook et al., 2014) and Paton et al. (2017) reported significant improvements in running economy and time to exhaustion in a BFR trained group relative to a non-BFR group (Paton et al., 2017) following four weeks of running training. The gained further improvements following adding BFR were ascribed to more muscular adaptation (Paton et al., 2017).

Improving both aerobic and anaerobic characteristics of players can be physically demanding and time-consuming in a separate session. Therefore, developing training methods which can simultaneously improve both physiological components using a single training mode is of great interest to coaches. Combining aerobic running training with BFR has been proposed as a single training method which has such multiple benefits (Abe et al., 2006, 2010; Ozaki et al., 2014; de Oliveira et al., 2016), thus, combining SSG with BFR can be a new approach that may assist futsal players to improve their fitness.

As the literature indicates, adding BFR to resistance and running training increases the internal exercise load which results in further muscle adaptations, although these kind of training train the main muscles in futsal, but they do not have the same involvement pattern in the futsal, thus considering the specificity principle of the training, we assumed that adding BFR to SSG would have more benefits. Therefore, the aims of this study were to investigate the effects of 3 weeks of game specific BFR training on specific performance of futsal players, and on changes in hormones (IGF-1 and MSTN) and neural indices related to potential strength gains.

## Materials and Methods

### Participants

The participants were 12 male futsal players who had played at least 5 years at Iran’s National League Second Division (age 23 ± 2 years, body height 174 ± 5 cm, body weight 67.5 ± 6.8 kg, body fat percentage 12.8 ± 2.1%, and BMI 22.2 ± 2.0 kg m^−2^). Exclusion criteria were more than one absence in a training session. All participants were healthy without any orthopedic, neuromuscular disorder or cardiovascular diseases and provided the informed consent for participation. Participants were randomly divided into two groups: futsal training with BFR (*n* = 6) and futsal training without BFR (non-BFR; *n* = 6). The study was approved by the Ethics committees of Sport Sciences Research Institute of Iran with code IR.SSRI.REC.1396.187.

### Procedures

**FIGURE 1:**
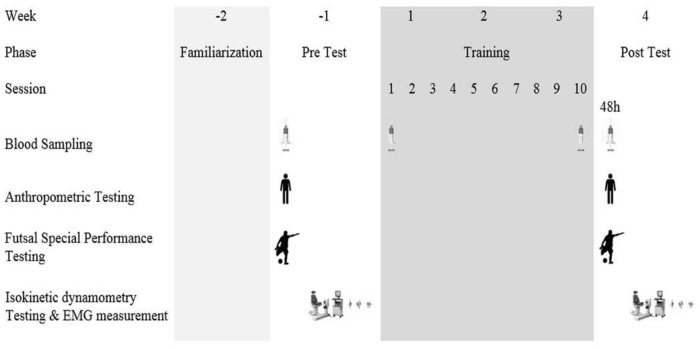
Schematic overview of study timeline.

The training protocol consisted of 10 sessions of 3-a-side game training (i.e., three players against three other players) played either with or without lower limb blood flow restriction. Figure 1 shows the testing timeline. Prior to and after the training period, a series of physiological tests was taken. Anthropometric characteristics such as body weight (digital weighing scales, Seca 769, Germany), body height (stadiometer, Seca 213, Germany), and body fat percentage (InBody S10, Biospace Co., Ltd., Seoul, Korea) were obtained.

### Isokinetic Strength

Isokinetic dynamometry (Isokinetic dynamometry, Biodex system 3, United States) was used to assess strength-related indexes. Participants sat on the chair of the Isokinetic dynamometry and attachments for leg extension were fixed to the dynamometer for preferred leg. Each participant did a number of knee flexion-extension at 60°s^−1^ in order to familiarize with isokinetic dynamometry. Strength parameters of quadriceps and hamstring muscles were recorded during five repetitive leg extension and flexion movements at a speed of 60°s^−1^ and Coefficient of Variation (CV) was set under 15% for knee extension and flexion. Peak torque and peak torque to body weight for leg extension and flexion were extracted from isokinetic dynamometry for further analysis.

### Electromyography

Electromyography signals of the m. vastus lateralis, m. vastus medialis and m. rectus femoris were registered simultaneously with an eight channel wireless Myomuscle Noraxon Electromyography system (NORAXON, United States) using 10 mm diameter adhesive electrodes at a frequency of 1500 Hz. The preparation of skin (i.e., shaving and rinsing with alcohol) surface was done to put the EMG electrodes according the SENIAM guidelines. Electrodes were connected to wireless EMG sensors (DTS EMG sensors; Noraxon) by EMG pinch lead wires (CMRR > 100 dB, Gain: 500). The signals were transmitted to a 16-bit analog to digital (A/D) converter receiver (Noraxon DTS receiver) and saved to a computer at a rate of 1500 Hz using the MR3 software (Version 3.10, Noraxon). Electromyography data were band pass filtered by Butterworth (high pass of 15 and low pass of 450 Hz) and the iEMG of all muscles was calculated for all repetitions.

### Futsal Special Performance Test

Futsal special performance test (FSPT) assess the performance and skill of futsal players. This test consists of nine steps include running with the ball, dribbling, turning, long and short passes, receiving a pass, performing a wall pass, shooting and running fast without the ball. Time was measured for each step and total time calculated. The test was carried out in accordance with the recommendations of Farhani et al. (2019).

### Training and Blood Flow Restriction Protocol

The training protocol utilized a high intensity 3-a-side futsal game played in a 20 × 20 m futsal court, for periods of 3 min activity and 2 min rest, the game was free touch and the ball was always made available promptly when it went out of play (Table 1; Owen et al., 2012). Both groups performed the game specific training but only one group performed training using BFR. Pneumatic cuffs (a 13 × 124 cm, Ghamat pooyan, Tehran, Iran) were worn by the BFR group on the upper part of both thighs during the training sessions. In the first week, cuffs were inflated to 110% of leg’s SBP during each 3 min exercise period and deflated in the resting. For the individualized pressure, a percentage of leg systolic blood pressure was used. Leg’s SBP is about 1.2-fold of arm SBP (Sareen et al., 2012). Pneumatic cuffs connected to gauge, after inflating and reaching to target pressure the connection was cut off. Players sat down on a bench and inflated the cuff, when the target pressure. When the target pressure was reached, players did three squats to ensure that the exact pressure was reached and then by closing the screw, the gauge was cut off. The pressure progressively increased by 10% mmHg after each two completed training sessions, except in the last session where the pressure was dropped as in the first session. HR (Polar RC3, Polar Electro Oy, Kempele, Finland) and the rate of perceived exertion (RPE, Borg scale CR-20) were recorded during all training sessions.

**Table 1:** Training protocol for two group, BFR section only for BFR group.

	Sessions 1–3	Sessions 4 and 5	Sessions 6 and 7	Sessions 8 and 9	Session 10
Drill	3 v 3	3 v 3	3 v 3	3 v 3	3 v 3
Exercise time (min)	3	3	3	3	3
Rest time (min)	2	2	2	2	2
Frequency	4	6	6	8	4
Intensity (%HRmax)	80–100	80–100	80–100	80–100	80–100
BFR (%SBP)	110	120	130	140	110

*%HRmax, percentage of maximal heart rate; %SBP, percentage of leg systolic blood pressure.*

### Blood Sampling and Analysis

A 5 cc blood sample was taken from the antecubital vein and collected in ethylenediaminetetraacetic (EDTA) tubes at four time periods. Samples were taken following an overnight fast, immediately after the first training session, immediately after the last training session and 48 h after the last session. Blood samples were centrifuged (4°C, 3000 rpm) for 10 min to isolate the serum, and then stored at −20°C and serum samples were analyzed for IGF-1 and myostatin. Serum concentration of IGF-1 was measured by Enzyme immunoassay (IGF-1 kit IBL, MD58011, Hamburg, Germany). The inter- and intra- assay variance were <10%. Serum concentration of myostatin was measured using enzyme immunoassay (MSTN kit CUSABIO, CSB-E11300h, Houston, United States). The inter- and intra assay variance were <15%.

### Statistical Analyses

Data are presented as mean ± SD. The normality of data was confirmed using the Shapiro-Wilk’s test. Independent paired sample *t*-tests and repeated ANOVA were performed to analyses differences within and between groups, respectively. Significance based analyses were performed using SPSS statistical software, version 19 for windows and the significant level was set at *P* = 0.05. Additionally, changes due to training within and between groups were reported using the effect size statistic (*D*) in accordance with the recommendations of Cohen (1988). The effect size was determined as the standardized difference between means divided by the pooled SD (Cohen, 1988). The effect size statistic was interpreted using the following criteria: trivial (<0.20), small (0.20–0.49), moderate (0.50–0.79) and large effects (>0.80).

## Results

### RPE and Heart Rate

The scores for RPE and HR during training were significantly greater (*p* ≤ 0.01) in the BFR group than non-BFR group. RPE values for non-BFR group were somewhat hard (13–14) and for another group was toward hard and very hard (15–17). The differences between the two groups were significant (*p* < 0.05). HR was significantly greater (171 ± 4 versus 166 ± 5 beats/min) in the BFR group than non-BFR group.

### Muscle Strength

The peak torque increased significantly (*p* = 0.01) for knee extension (30.9 ± 8.0 versus 14.9 ± 7.5%) and flexion (23.8 ± 8.4 versus 8.1 ± 5.7%) in both groups with significant differences between groups (*p* = 0.01) favoring the BFR group and these were considered a large effect (knee extension, ES = 1.3; knee flection, ES = 1.0) (Table 2). The iEMG of m. vastus lateralis, m. vastus medialis, and m. rectus femoris increased significantly in both groups (*p* = 0.01) and there was only a significant difference (*p* = 0.02) between groups in m. rectus femoris in favor of the BFR group and this was considered a large effect (ES = 1.1).

**Table 2:** Strength and muscle activation (pre- and post-training) for BFR and non-BFR groups.

Variables	BFR			Non-BFR			Group difference	
	
	Pre-training	Post-training	Changes [±95%CI]	Pre-training	Post-training	Changes [±95%CI]	*P* value	ES [±95%CI]
Record of FSPT (s)	34.9 (2.5)	30.4^∗^ (2.6)	−11.0 [4.6]	33.8 (3.1)	31.8 (3.2)	−6.0 [17.4]	0.45	−0.6 [1.5]
PT of knee extension (Nm)	185.8 (22.0)	242.0^∗^ (12.7)	30.9 [7.9]	190.9 (20.6)	218.5^∗^ (9.2)	14.9 [7.5]	0.01#	1.3 [0.8]
PT of knee flexion (Nm)	83.2 (13.1)	102.3^∗^ (9.6)	23.8 [8.4]	81.4 (8.3)	87.7^∗^ (5.3)	8.1 [5.7]	0.01#	1.0 [0.6]
iEMG of vastus lateralis (mv)	2919.3 (893.8)	4140.3^∗^ (1085.2)	42.47 [18.40]	2699.8 (553.8)	3464.5^∗^ (983.3)	25.9 [14.8]	0.19	0.5 [0.6]
iEMG of vastus medialis (mv)	2632.0 (1006.5)	3531.7^∗^ (1070.6)	33.8 [15.9]	2832.5 (1346.4)	3585.0^∗^ (1047.9)	32.8 [24.3]	0.94	0.1 [0.5]
iEMG of rectus femoris (mv)	3123.2 (686.2)	4985.0^∗^ (1004.7)	60.5 [24.2]	3003.0 (573.6)	3639.3^∗^ (998.8)	19.0 [11.3]	0.02#	1.1 [0.7]

*BFR, blood flow restriction; FSPT, Futsal Special Performance Test; S, Second; PT, Peak Torque; iEMG, integrated Electromyography; Nm, Newton meter; mv, millivolt. Date are mean (SD). “#” indicates difference between groups. “^∗^” indicates difference within the group.*

### Futsal Special Performance Test

Performance time for the FSPT decreased significantly in BFR group (*P* = 0.001) by −11.0% (ES = −1.38) and non-significant in non-BFR group (*p* = 0.381) by −6.0% (ES = −0.58) respectively following training. However, there was no significant differences between groups (*p* = 0.45).

### Hormones

In comparison to baseline, there were no significant differences in the levels of IGF-1 at any measured time points. Also, MSTN levels did not change significantly in response to the game play in either group (Table 3).

**Table 3:** Data of hormones in BFR and non-BFR groups.

Variables	Group	Baseline	Acute 1	Acute 2	Adaptation
IGF-1 (ng/mL)	BFR	116.1 (2.8)	117.0 (6.2)	118.15 (4.5)	116.1 (4.7)
	Non-BFR	116.1 (5.0)	114.7 (2.8)	117.3 (5.9)	117.8 (5.9)
MSTN (ng/mL)	BFR	10.2 (1.1)	10.8 (1.1)	10.2 (1.4)	8.3 (1.1)^∗#^
	Non-BFR	10.3 (1.5)	10.8 (1.3)	10.3 (2.2)	10.7 (1.2)
IGF-1/MSTN	BFR	11.3 (1.2)	11.1 (1.0)	11.7 (1.7)	14.1 (1.8)^∗#^
	Non-BFR	11.4 (1.5)	10.7 (1.4)	11.9 (2.6)	11.1 (1.1)

*BFR, blood flow restriction; baseline: before getting pre-testing, Acute1, immediately after first session; Acute 2, immediately after last session; adaptation: 48 h after last training session. Date are mean (SD). “^∗^”Intragroup significant differences with Baseline at p < 0.05; “^#^”intergroup significant differences at p < 0.05.*

After completing the training protocol, the resting levels of MSTN decreased, significantly in the BFR group (*p* = 0.01); the difference between groups was significant (*p* = 0.01, ES = −1.51). Accordingly, the resting post-training IGF-1/MSTN ratio increased, significantly in the BFR group (*p* = 0.01); and the intergroup difference was significant (*p* = 0.01, ES = 1.63) (Table 3).

## Discussion

The study aimed to identify the effects of a 3-week (10 sessions) SSG training combined with BFR on specific performance of futsal players and also the potential role of IGF-1 and MSTN on strength gains and performance in specific tasks of futsal. The results show that applying BFR increases the internal load of the training (HR and RPE) and it results in the small additional enhancements in several aspects of muscle activation in comparison with the same exercise without BFR. The time for the FSPT improved in both groups but there was no significant difference between groups. At measured time points, only 48 h after the last training session, the level of MSTN decreased in the BFR group, which appears BFR training leads to a reduction in hypertrophic inhibitory factors initially.

The physiological stress of exercise measured with RPE and HR was higher in BFR group than the non-BFR group. It is probable that the mean of RPE scores in the BFR group were higher than the other group because of the lower oxygen conditions, muscle swelling due to greater metabolite stress and pain of cuff pressure, in fact, the RPE values were rated fairly light to somewhat hard for non-BFR group and for BFR group, they were hard and very hard. Consistent with previous BFR studies, HR in the BFR group was higher than non-BFR group; it was assumed that this was due to increased compensation pooling the venous blood in distal of occlusion point (Pope et al., 2013).

A bout of BFR resistance exercise like high-intensity resistance exercise appears to stimulate similar increases in anabolic and catabolic hormone responses (Kim et al., 2014). However, to our knowledge, there are a no studies investigating hormonal responses carried out during running-based exercises with blood flow occlusion. It is thought cell swelling and metabolites accumulation may increase the anabolic response by releasing GH (Loenneke et al., 2012a). GH has shown to be stimulated by an acidic intramuscular environment (Takarada et al., 2000b) and also evidence points out that a low pH stimulates sympathetic nerve activity through group III and IV afferent fibers which plays an important role in the regulation of hypophyseal secretion of GH (Loenneke et al., 2010). Although GH stimulates the release of IGF-1 (Kraemer and Ratamess, 2005), but no significant changes in IGF-1 levels were seen in response to the game, though timing of blood sampling (immediately after) may be involved in this response. It has been shown that there was a non-significant increase in IGF-1 levels immediately after the heavy resistance exercise and it started to decrease over time (Hasani-Ranjbar et al., 2012). Since IGF-1 is involved in the glucose homeostasis (Clemmons, 2004), so this non-significant changes in IGF-1 levels immediately after a 3-side game might be a compensatory mechanism for preventing the post-exercise hypoglycaemia. Our results showed that in both groups, serum MSTN levels did not change in acute response to the game; whereas it has previously been shown that after an acute running and resistance exercise, MSTN mRNA was decreased significantly 1 h after exercise (Louis et al., 2007), thus it takes time to express the protein at the serum level, so time of blood sampling may be critical.

There were no significant changes in the post-training level of IGF-1 and GH in both groups, but a significant decrease was observed in serum MSTN post-training level in the BFR group and this finding has been supported by previous resistance BFR studies (Drummond et al., 2008; Laurentino et al., 2012) and aerobic training studies without BFR (Hittel et al., 2010). MSTN is a down regulator of muscle growth which may be influenced by a greater distribution of fast-twitch muscle fibers (Matsakas et al., 2006), so since BFR exercise recruited the type II muscle fibers, after the 3 weeks futsal game training with BFR, decreased levels of MSTN were expected. Thus, it could be stated that during the BFR training, decreasing the levels of proteins involved in muscle breakdown occurs earlier than the proteins involved in muscle synthesis.

In the present study, quadriceps strength increased significantly in both groups, but the magnitude of the observed strength gain in the BFR group was greater than the non-BFR group. The strength changes in response to BFR training thought to depend on both muscle hypertrophy and non-hypertrophy mechanisms. The two major mechanisms thought to be responsible for provoking skeletal muscle adaptation following BFR are cell swelling (Loenneke et al., 2012a) and metabolite accumulation (Jessee et al., 2018) which inhibit protein breakdown or increase protein synthesis. The accumulation of metabolites during BFR may indirectly stimulate anabolic hormonal pathways (Loenneke et al., 2012a), by stimulating the group III and IV afferent fibers (Pope et al., 2013) which in turn leads to further recruitment of fast twitch fibers, resulting in a hypertrophic mechanical stimulus as well as strength increment.

Although, no metabolites accumulation has been reported in previous BFR walking study (Loenneke et al., 2012b), the exercise intensity in our study was much greater and likely lead to high metabolites accumulation. In the study, no significant changes in the resting post-training level of IGF-1 and GH and a significant decrease in MSTN were observed, thus increased strength may be attributable to a non-hypertrophic mechanism like increased recruitment of fast twitch fibers which may be inferred from the iEMG results. The iEMG results showed that there were significant increases in muscle activation of the quadriceps’s iEMG in both group favor to BFR group so that it significantly increased in BFR group than non-BFR for rectus femoris. The increase in iEMG can be attributed to an increase in motor unit recruitment in BFR groups. These results can also be confirmed by the results of peak torques of quadriceps during isokinetic testing, in which there were significant increases in peak torque of knee muscles in BFR groups than non-BFR group. Increased metabolite accumulation with BFR leads to greater recruitment of type II fibers (Loenneke et al., 2015) and may hasten the fatigue and consequently increase the number of motor units recruited (Yasuda et al., 2014; Loenneke et al., 2015) which will in turn increase iEMG amplitude. Brandner et al. (2015) found following BFR exercise motor-evoked potential lasted up to 1 h following exercise, so following BFR training, the increased excitability of central motor pathways could lead to greater long-term adaptations (enhance strength) which in motor unit recruitment pattern might be altered (Brandner et al., 2015). Thus, it seems that type III and IV sensory fibers which stimulated by the metabolite accumulation during BFR are involved so, metabolic stress plays the most prominent role in instigating the muscle adaptations (Suga et al., 2012).

Although training with BFR offers several positive benefits, we acknowledge that there were several limitations of this study. The sample size was small because of the number of players in the team and the individual response to training may effect on results. Also, it was our thought that high pressure could limit futsal activities, but in practice there was not any limitation to do them.

## Conclusion

In conclusion, our findings suggest that the addition of BFR to SSG can provide greater physical enhancements specifically at the muscle activation and endocrine level. Fitness coaches could apply the BFR to impose more physical stress on players in order to gain increased training benefits.

## Data Availability

The datasets generated and analyzed during the current study are available from the corresponding author on reasonable request.

## Ethics Statement

All procedures performed in studies involving human participants were in accordance with the ethical standards of Sport Sciences Research Institute of Iran with code IR.SSRI.REC.1397.187 and with the 1964 Helsinki declaration and its later amendments or comparable ethical standards. This article does not contain any studies with animals performed by any of the authors.

## Author Contributions

SA-S, HR, and CP conceived the study. FF, AA, AS, and SA-S conducted the experiments. SA-S, CP, and MB analyzed the study. SA-S, HR, CP, MB, DB-d-F, TR, PN, and BK interpreted the data for the study. All authors made substantial contributions to the design of the work, drafted the work or revised it critically for important intellectual content, provided final approval of the version to be published, and agreed to be accountable for all aspects of the work in ensuring that questions related to the accuracy or integrity of any part of the work are appropriately investigated and resolved.

## Conflict of Interest Statement

The authors declare that the research was conducted in the absence of any commercial or financial relationships that could be construed as a potential conflict of interest.

## Abbreviations

BFR: blood flow restriction
EMG: electromyography
ES: effect size
FSPT: futsal Special Performance Test
GH: growth hormone
HRmax: maximum heart rate
iEMG: integrated electromyography
IGF-1: insulin growth factor-1
MSTN: myostatin
RPE: rate perceived exertion
SBP: systolic blood pressure
SSG: small sided games
VO_2_max: maximum oxygen consumption.
